# Hospital admission patterns subsequent to diagnosis of type 1 diabetes in children : a systematic review

**DOI:** 10.1186/1472-6963-7-199

**Published:** 2007-12-05

**Authors:** Val C Angus, Norman Waugh

**Affiliations:** 1 College of Life Sciences and Medicine, University of Aberdeen, West Block, Polwarth Building, Foresterhill, Aberdeen, AB25 2ZD, UK; 2 Department of Public Health, University of Aberdeen, Polwarth Building, Foresterhill, Aberdeen, AB25 2ZD, UK

## Abstract

**Background:**

Patients with type 1 diabetes are known to have a higher hospital admission rate than the underlying population and may also be admitted for procedures that would normally be carried out on a day surgery basis for non-diabetics. Emergency admission rates have sometimes been used as indicators of quality of diabetes care.

In preparation for a study of hospital admissions, a systematic review was carried out on hospital admissions for children diagnosed with type 1 diabetes, whilst under the age of 15. The main thrust of this review was to ascertain where there were gaps in the literature for studies investigating post-diagnosis hospitalisations, rather than to try to draw conclusions from the disparate data sets.

**Methods:**

A systematic search of the electronic databases PubMed, Cochrane LibrarMEDLINE and EMBASE was conducted for the period 1986 to 2006, to identify publications relating to hospital admissions subsequent to the diagnosis of type 1 diabetes under the age of 15.

**Results:**

Thirty-two publications met all inclusion criteria, 16 in Northern America, 11 in Europe and 5 in Australasia. Most of the studies selected were focussed on diabetic ketoacidosis (DKA) or diabetes-related hospital admissions and only four studies included data on all admissions. Admission rates with DKA as primary diagnosis varied widely between 0.01 to 0.18 per patient-year as did those for other diabetes-related co-morbidity ranging from 0.05 to 0.38 per patient year, making it difficult to interpret data from different study designs. However, people with Type 1 diabetes are three times more likely to be hospitalised than the non-diabetic populations and stay in hospital twice as long.

**Conclusion:**

Few studies report on all admissions to hospital in patients diagnosed with type 1 diabetes whilst under the age of 15 years. Health care costs for type 1 patients are higher than those for the general population and information on associated patterns of hospitalisation might help to target interventions to reduce the cost of hospital admissions.

## Background

Type 1 diabetes mellitus (T1DM), (International Classification of Diseases, ICD-9 codes 250.0 to 250.9; ICD-10 codes E10 to E14), formerly known as insulin-dependent diabetes mellitus (IDDM), is an endocrine disorder that is associated with a wide range of co-morbidities such as heart disease, kidney disease, eye problems and foot problems.

Children with T1DM can produce little or no insulin, which is essential to sustain life, and require a package of care involving insulin injections, usually several times a day, self-blood glucose testing and adjustments of insulin dose and diet according to levels of physical activity. Trials such as the Diabetes Control and Complications Trial (DCCT, [1]) have shown that good control, as reflected in glycosylated haemoglobin (HbA1c), reduces complication rates and should reduce the need for hospital admissions for diabetic complications such as diabetic ketoacidosis (DKA), which is an important cause of morbidity and occasional mortality in children with T1DM. One might expect that changes in the intensity of insulin therapy in the years since the DCCT results were published, might have reduced admission rates.

The objective of this review was to survey studies of hospital admission patterns, whether diabetes-related or non-diabetes related, in patients diagnosed with T1DM whilst under the age of 15. The review was designed to identify gaps in the literature as a precursor to carrying out a study of all hospitalisations for a Scottish population-based incidence cohort diagnosed with type 1 diabetes whilst under the age of 15. Hospital admission rates have the potential to be utilised as an indication of the efficacy of different treatment programmes, and can also be a key element in assessing the full cost of diabetes. Studies of admission at diagnosis were not included, having been the subject of a Cochrane review by Clar and colleagues [2]. The limitation to those diagnosed under 15 years of age was because this review is a prelude to a record linkage exercise using the register of the Scottish Study Group for the Care of Diabetes in the Young (SSGCDY), which only includes under 15s.

## Methods

### Search strategy

A bibliographic database search for literature published over the last 20 years was conducted to retrieve publications relating to hospitalisation of T1DM cohorts. Searches were made of the following electronic databases: PubMed, Cochrane Library, MEDLINE, and EMBASE for the period 1986 to 2006, limited to English language. Research by Royle, Bain and Waugh [3,4] confirmed that an electronic search of these databases would ensure comprehensive coverage of the available literature in diabetes epidemiology.

An initial broad search was undertaken for publications referring to hospitalisation of patients with T1DM using the following search criteria:

A in title AND B in title or keyword

where dollar ($) is a wild card allowing the use of word stems

A = Hospital Admissions

"admission$" OR "hospital$" OR "ketoacidosis" OR "DKA"

B = Type 1 Diabetes

"IDDM$" OR "T1DM" OR "TIDM" OR "insulin-dependent"

OR "diabet$" in title AND ("type 1" OR "type I") as keyword

All references cited by the selected publications were also examined for inclusion in the literature search and a direct search made of the main diabetes journals. After the removal of duplicates, this search yielded 416 separate publications.

### Inclusion and exclusion criteria

For each publication retrieved from the electronic search, the titles and abstracts were initially assessed according to specific inclusion and exclusion criteria (Table 1).

**Table 1:** Inclusion and exclusion criteria

**Selection**	**Inclusion**	**Exclusion**
**Study population**	Children diagnosed with T1DM whilst under the age of 15 years old	No separate data on children diagnosed with T1DM.
**Study Design**	All study designs	Studies focussed on specific non-diabetic co-morbidities
**Data Collection**	Post-diagnosis hospital discharge data analysed	No data on post-diagnosis hospitalisation
**Outcome**	Hospitalisation rates for all diagnoses and co-morbidities.	Main focus on rates for type 2 diabetes mellitus

Full text papers were examined in cases where the abstract did not provide sufficient information to be able to make a judgement on inclusion. All publications relating to diabetic adults only or not categorising T1DM separately from type 2 were excluded, as were publications focussing on incidence of diabetes only, with no mention of subsequent hospitalisation or follow-up.

## Results

### Review of Literature – Overview

**Figure 1:**
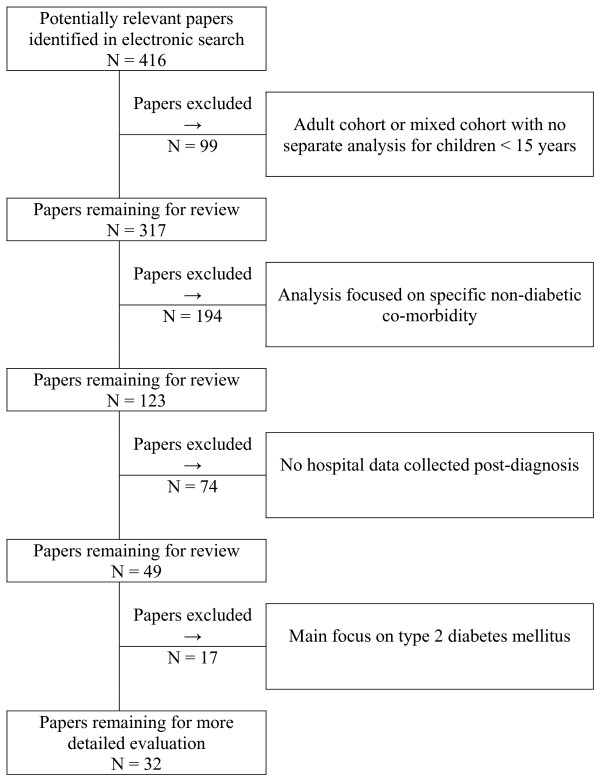
Exclusion of papers extracted in literature search.

During assessment, the overall number of publications suitable for inclusion decreased from 416 to 32, the selection process is illustrated in Figure 1.

The remaining publications were published in 15 different journals, primarily Diabetes Care (9, 28%), Pediatrics (4, 13%) and Diabetic Medicine (3, 9%). Most of the selected publications were not focussed on all categories of post-diagnosis hospital admissions for T1DM children, but all included at least some information on aspects of hospitalisation in T1DM children, post-diagnosis. Although there was a certain amount of overlap, the main focus of the selected publications centred on one of the following three categories:

A) Hospital admissions for T1DM patients (15 papers)

B) DKA and subsequent DKA admissions (11 papers)

C) Hospital admission rates following different treatment programs (6 papers)

All of the 32 publications in these categories mentioned subsequent hospitalisation of children following a previous diagnosis of T1DM and are described in the following subsections, followed by a summary of research relating to hospitalisation of T1DM children post-diagnosis.

For each category, a summary table of the selected publications is provided, detailing the main characteristics of the publication. Where possible, study size has been given as the post-diagnosis T1DM cohort corresponding to re-admissions and excludes new admissions at diagnosis and older age groups if they have been analysed separately.

Most papers provided rates in terms of admissions per patient year for the T1DM cohort, but a minority of papers expressed rates in terms of admissions per 100,000 population or as a ratio of T1DM admission rates to entire population admission rates and did not provide the underlying admission rate for the population, such that hospitalisation rates for the T1DM cohort could be inferred. These anomalies are noted in the summary table.

### Hospital admission for T1DM patients

The main focus for papers in this section is hospitalisation for T1DM patients diagnosed in childhood. Most of the publications only include data on hospital re-admissions with a discharge code for diabetes or DKA, but a few publications did include co-morbidities. A summary table of the 15 publications selected for further review is provided in Table 2.

**Table 2:** Studies reporting all admissions

**Author**	**Country**	**Study Design**	**Study Size**	**Age Group**	**Admission Rate/patient-year**	**Comments**
Charron-Prochownik 1993 [11]	USA	Longitudinal cohort study following up IDDM cases recruited 1981 – 84	88	8–13	0.10	Analysis of admissions coded as diabetes only
Cohn 1997 [18]	USA	Retrospective analysis of IDDM admissions 1985 – 99	2889	0–18	0.10 – 0.38	Principal diagnosis of IDDM. Female excess of IDDM re-hospitalisations
Donnan 2000 [16]	Scotland	Retrospective cohort study for admissions in 1995	864	0–35	Relative Risk 2.89 cf general population	Combined age group for T1DM and no direct hospitalisation rates
Hirasing 1996 [10]	Holland	Retrospective analysis of T1DM admissions 1980 – 81	Not given	0–19	0.24 – 0.41	Diabetes-related admissions only Including diagnosis
Icks (a) 2001 [15]	Germany	Case-control study of T1DM cases diagnosed between 1996 – 97, with 1.5 years follow-up	373	1–14	0.34	All hospital admissions. 4.7 times higher risk of hospitalisation than controls
Icks (b) 2001 [14]	Germany	Prospective analysis of admission records for T1DM children diagnosed in 1997	5874	1–19	0.27	All admissions. 3 times higher risk of hospitalisation than general population
Lipton. 2002 [13]	USA	Retrospective T1DM cohort study of risk factors for re-hospitalisation	216	0–18	0.23	Diabetes-related admissions only
Moss 1999 [6]	USA	Population study Follow-up at 4 and 10 years	777	0–30	>0.26	All admissions. Hospitalisation prior to examination
O'Hara 1998 [9]	Australia	Retrospective analysis of admission records 1993 – 95	95091	1–75+	Not supplied	All diabetes-related admissions at diagnosis and after
Palta 1996 [7]	USA	Retrospective follow-up of T1DM cohort diagnosed 1987 – 92	507	0–19	0.05 – 0.20	All reported diabetes-related hospital admissions
Palta 1997 [8]	USA	Follow-up of recruited T1DM cohort diagnosed 1987 – 92	577	0–29	0.07 – 0.10	All reported diabetes-related hospital admissions
Roberts 2004 [19]	England	Retrospective analysis of T1DM cohort diagnosed 1968 – 96 with 3-year follow-up	Not given	0–29	4992 admissions over 3 years	Diabetes-related admissions only Focus on mortality in young people
Sutton 1989 [5]	Australia	Retrospective analysis of IDDM cohort diagnosed 1985 – 87	650	0–19	0.06	Diabetes-related admissions only
Tomlin 2006 [17]	New Zealand	Case-control study comparing admission rates between diabetic and non-diabetic children 2000 – 02	1123	0–14 15–24 25–34 ---- 85+	0.65 0.40 0.40 0.62	All admissions. T1DM patients 2.55 times more likely to be admitted to hospital than general population
Tuomilehto. 1997 [12]	Finland	Retrospective follow-up of IDDM cohort diagnosed 1965 – 1979	5149	0–18	0.01	Hospitalisation for diabetic nephropathy only

Amongst 650 IDDM children in Sydney, the rate of post-diagnosis admissions was 0.55 per patient-year in 1987 [5]. The majority of admissions were due to poor control of diabetes. High glycosylated haemoglobin levels and hypertension were identified by Moss *et al*. [6] as predictive factors for hospitalisation for 777 diabetic patients under the age of 30, living in Wisconsin. High HbA1c indicates poor glycaemic control, and hypertension often accompanies progressive renal disease, increasing the risk of myocardial infarction and stroke. Participating patients were followed up at 4 and 10 years and asked to provide details of any hospitalisations in the preceding year. The results for the 10 years following diagnosis indicated average hospitalisation rates of 0.26 per patient-year, but this figure only included the first admission per patient, whereas other studies included all yearly admissions in hospitalisation rates. Palta *et al*. [7] also focussed on glycaemic control, and as part of their analysis, reported the rate of diabetes-related hospitalisation of T1DM children in Wisconsin under the age of 20 as ranging from 0.05 and 0.20 per patient-year, again being correlated with glycaemic control. Poor glycaemic control as a risk factor was also highlighted the following year when Palta *et al*. [8] completed a further study focussing on hospitalisation of a similar cohort of 577 T1DM children, estimating 0.09 per patient-year as the diabetes-related admission, with females and children in the 12–16 age category being most at risk of re-hospitalisation. They also examined diabetes-related hospitalisation rates for each year following diagnosis, which showed the lowest rate (0.07 per patient-year) in year 6 and the highest rate in years 1 and 5 (0.10 per patient-year). However, the cohort used in this study was recruited from volunteers and may not be representative of the underlying population, although uptake rate was fairly high (79%).

Hospital admission for complications of diabetes in Victoria, Australia over a 2-year period was investigated by O'Hara and McCarty [9], but this data was not correlated with the T1DM cohort in the population, did not differentiate between first and subsequent hospital admissions and focussed on diabetic complications for all age groups. The study by Hirasing *et al*. [10] extracted all diabetes-related hospitalisations for Dutch children aged 0–19 years old in 5-year age bands, but they were unable to differentiate between admissions at diagnosis and re-admissions and only provided indirect hospitalisation rates estimated from the underlying population and the prevalence of T1DM cases. However, the study did provide some evidence for an overall reduction in hospital admission rates between 1980 and 1991 falling from 0.41 to 0.24 per patient-year, but this could be biased by the inclusion of new admissions at diagnosis, which have reduced over time following an initiative to promote home instead of hospital care at diagnosis. The appointment of additional diabetes specialist nurses has enabled more T1DM children to be cared for at home as opposed to hospital.

A small follow-up study of 88 children aged between 8 and 13, carried out in Pittsburgh over a two year period, identified a re-admission rate of 0.10 per patient-year for any admission with a discharge code for diabetes [11]. This study focussed on psychosocial, demographic and biomedical predictors of early re-hospitalisation and found an increased risk of re-admission following initial diagnosis, for younger children, cases with a lower socio-economic status and cases with severe symptoms or higher levels of glycosylated haemoglobin at diagnosis.

A study by Tuomilehto *et al*. [12] reported admissions for kidney disease (diabetic nephropathy) for 5149 IDDM children in Finland, diagnosed for type 1 diabetes whilst under the age of 18. They linked the Finnish IDDM register to routine hospital discharge diagnoses. This study found an overall increasing trend in the incidence of diabetic nephropathy over a 24 year follow-up period, rising from 0.01 per patient-year after 10 years of follow-up and peaking at 0.03 per patient-year after 20 years.

Lipton *et al*. [13] examined risk factors for 216 African-American and Latino children diagnosed with T1DM under the age of 18 years and followed up for approximately 6 years. Their study showed that the diabetes-related hospitalisation rate was in the region of 0.23 per patient-year and that females were twice as likely to be hospitalised as males. A study of 5,874 German T1DM children aged 1–19 years old also found a higher hospitalisation rate for females, but to a much lesser extent 0.29 per patient-year for girls compared to 0.26 for boys and a 3-fold increase in admissions for diabetic children compared with the general population [14]. Icks *et al*. [15] also conducted a smaller case-control study of 373 T1DM patients, diagnosed between 1996 and 1997, aged between 1 and 14 years and 783 age/sex-matched non-diabetic controls. This study found hospitalisation rates of 0.34 per patient-year for T1DM children and 0.07 in the non-diabetic controls – a five-fold increase and an eight-fold increase in the length of hospital stays.

In a study of T1DM patients, resident in Tayside and diagnosed between 0 and 35 years old, Donnan, Leese and Morris [16] found that the annual hospital admission rate for T1DM patients in 1995 was nearly three times that of the local population, although length of stay in hospital was comparable. The younger age groups were not analysed separately. More recently, a case control study carried out in New Zealand by Tomlin *et al*. [17] examined rates of admission for 12,458 diabetic patients (type 1 and type 2) and non-diabetic controls over a period of 3 years. A small proportion of patients (1123, 9%) in the 0 to 43 age group were categorised as T1DM, but hospitalisation rates were provided for young age groups. Results for the 0–14 age group (the majority of whom will be T1DM) indicated a hospitalisation rate of approximately 0.10 per patient-year for non-diabetic controls, 0.50 per patient-year for diabetic patients with diabetes-related complications and 0.65 per patient-year for diabetics admitted to hospital with any diagnosis.

In California, a study by Cohn *et al*. [18] found increased hospitalisation rates for young girls compared to boys in a cohort of 2,889 IDDM children aged 0–18 years during 1991. This was particularly high for girls in the 15–18 age group, (0.27 per patient-year for boys and 0.38 per patient-year for girls), but the difference may have been due to eating disorders in young girls in this age group.

Mortality in young T1DM people admitted to hospital in Oxford was the main focus of a publication by Roberts, Goldacre and Neil [19]. They found that the death rate for the T1DM cohort within three years of hospital admission was nine times higher than in the general population. Although this paper provided the number of diabetic admissions over the 3-year follow-up period, no information was provided on the underlying T1DM population to enable the calculation of hospitalisation rates post-diagnosis.

### DKA and subsequent DKA admissions

Publications in this section focus on diabetic ketoacidosis (DKA) and/or subsequent hospitalisations after the initial diagnosis of T1DM. None of these publications provide hospitalisation rates for other co-morbidities except for DKA. A summary table of the 11 publications selected for review is provided in Table 3.

**Table 3:** Studies reporting admissions for DKA

**Author**	**Country**	**Study Design**	**Study Size**	**Age Group**	**Admission Rate/patient-year**	**Comments**
Bui 2002 [21]	Australia	Retrospective analysis of DKA admissions 1985 – 1999	332	0–19	0.33	Diabetes-related hospitalisations DKA associated with adverse metabolic events, cerebral edema and death
Curtis 2002 [20]	Canada	Retrospective analysis of diabetes-related admissions 1991 – 2000	15872	0–18	52 – 70 per 100,000 population	Hospitalisation rates calculated for whole population. DKA rates stable
Dunger 2004 [25]	Europe and North America	Consensus statement on DKA in children and adolescents based on literature review	Many studies	0–19	0.01 – 0.10	DKA admission rates only
Jiang 2003 [29]	USA	Retrospective study of diabetes-related hospital admission records	4698	1–17	Not supplied	Multiple hospitalisation data not provided for estimating hospitalisation rates
Keenan 2002 [24]	USA	Retrospective analysis of T1DM cohort for diabetes-related admissions 1996 – 1997	8566	1–20	Not supplied	DKA and diabetic coma admissions only Focus on costs No admission rates presented
Kovacs 1994 [30]	USA	Longitudinal 14-year follow-up of recruited IDDM cases diagnosed 1978 – 1985	95	8–13	0.15	DKA admission rates only
Morris 1997 [27]	England	Retrospective cohort analysis of IDDM patients attending clinic 1993 – 1994	89	1–29	Not supplied	DKA admission rates and diabetic complications only Focus of correlation with HbA_1C _levels.
Rewers 2002 [23]	USA	Prospective follow-up of a T1DM cohort 1996 – 2000	1243	0–19	0.08	DKA admission rates only
Rosilio 1998 [22]	France	National survey of T1DM children using 206 clinic centres	2579	1–19	0.01	DKA admission rates and severe hypoglycaemia events
Smalldone 2005 [28]	USA	Retrospective analysis of hospital records for T1DM cohort 1998 – 2000	Not given	0–18	Not supplied	Focus on comparison between single and multiple DKA admission rates
Smith 1998 [26]	England	6 year retrospective case review 1990 – 1996	135	0–18	0.10	DKA admission rates only

In Ontario, 15,872 children under the age of 19 were identified over a 9-year period with diabetes-related hospital admissions [20]. This study presented admission rates per 100,000 population and did not differentiate between new admissions and re-admissions for diabetic children. However, it did conclude that DKA admission rates remained stable between 1992 and 1999.

Bui, Werther and Cameron [21] ascertained admission trends for DKA in Victoria, Australia over a period of 15 years (from 1985 to1999), based on retrospective analysis of hospital admission records. They classified children as "relapsers" with two or more DKA episodes per year and "non-relapsers" with one DKA episode per year and found a stable rate of DKA admissions in "non-relapsers" and a downward trend in "relapsers", with an average DKA hospitalisation rate of 0.33 per patient-year. Girls were twice as likely as boys to be admitted for DKA.

Rosilio *et al*. [22] studied 2,579 French children with T1DM focussing on factors associated with glycaemic control, reporting hospitalisation rates as 0.45 per patient year for DKA and severe hypoglycaemia events. Similarly, Rewers *et al*. [23] studied a cohort of Denver children aged under 20 years to determine DKA rates and prediction factors. They observed a lower DKA hospitalisation rate of 0.08 per patient-year, which increased to 0.19 per patient-year when severe hypoglycaemia was included.

A larger study by Keenan, Foster and Bratton [24] determined that social factors were associated with an increased risk of hospitalisation for 8566 T1DM children in North Carolina, but their study concentrated on length of hospital stay and associated costs, rather than on specific post-diagnosis hospitalisation rates. Subsequently, the European Society for Paediatric Enocrinology (ESPE) in conjunction with Lawson Wilkins Pediatric Endocrine Society (LWPES) produced a comprehensive review on DKA in children and adolescents [25]. This review indicated that the risk of DKA admissions post-diagnosis for T1DM children was between 0.01 and 0.10 per patient per year. One of the referenced studies [26] estimated the risk at 0.10 per patient-year on the basis of a 6-year retrospective case note review of 135 diabetic children in Manchester and found that "the vast majority of ketoacidosis episodes in established diabetic children were caused by abnormal insulin treatment behaviour". This is in agreement with a study conducted in Dundee where adherence to insulin treatment protocols was found to be inversely related to hospital admissions for DKA suggesting that poor adherence to insulin treatment was a major factor in DKA hospitalisations [27]. A couple of years later, Smaldone *et al*. [28] examined DKA hospitalisations for Californian children under the age of 19. They compared the characteristics of children with single versus multiple DKA hospitalisations and found higher ratios in older children and females, but did not specifically provide hospitalisation rates or cohort size for the underlying T1DM population. Jiang *et al*. [29] also analysed multiple hospitalisations for adults and children separately, but did not present raw data for estimating re-admission rates, focussing more on costs. However, in a Pittsburgh study, Kovacs, Charron-Prochownik and Obrosky [30] did report DKA hospitalisation rates of 0.15 per patient-year for T1DM children with hospitalisations observed over a 14-year period. They focussed on predictors of multiple hospitalisations and concluded "the evidence indicates that early identification and intervention could reduce rates of overall admission as well as multiple hospitalizations among young people with IDDM".

### Hospitalisation rates for different treatment programs

The publications in this section compare hospitalisation rates in T1DM children as an outcome measure following different management programs. A summary table of the six publications selected for review is provided in Table 4.

**Table 4:** Studies reporting the effects of interventions

**Author**	**Country**	**Study Design**	**Study Size**	**Age Group**	**Admission Rate/patient-year**	**Comments**
**Randomised trials**						
Laffel 1998 [33]	USA	Randomised Control Trial of **IDDM **cases assigned to standard care or ambulatory care intervention	171	10–15	0.09 Treated 0.24 Standard	Admissions codes for diabetes only
Laffel 2006 [34]	USA	6-month Randomised Control Trial of IDDM cases assigned to either a blood ketone or urine ketone monitoring group	123	3–22	0.38 Blood 0.75 Urine	Admissions codes for diabetes only Also includes emergency assessments
**Non-randomised studies**						
Glasgow 1991 [31]	USA	Retrospective analysis of diabetes hospital admission records 1984 – 1988	267	0–20	0.14 – 0.26	Admissions codes for diabetes only
Levine 2002 [35]	USA	Prospective 1-year follow-up of T1DM cohort classified into one of 3 baseline HbA_1c _levels	300	7–16	0.13 Overall 0.11 <8.1% 0.04 8.1–9.0% 0.25 >9.0%	Admission rates three times rate in general population
Svoren 2003 [36]	USA	2-year prospective Randomised Control Trial of T1DM children assigned to 3 treatment groups	299	7–16	0.18 CA 0.09 CA+ 0.13 SC	CA – Ambulatory diabetes care CA+ – CA plus psychoeducational modules SC – Standard care
Swift 1993 [32]	England	Retrospective T1DM cohort study 1978 – 1988 of hospital admissions post-diagnosis over 10 years, comparing home and hospital management at diagnosis	236	10–14	0.02 Home 0.04 Hospital	Home – Initial management without hospitalisation Hospital – Initial hospital management

A new treatment plan introduced at a Washington hospital utilised a diabetes team to ensure that new patients consistently received insulin injections [31]. This new programme resulted in a significant 47% reduction in re-admission rates over a 5-year period from 1984 to 1988 (0.26 to 0.14 per patient-year, p < 0.05), even though there was a large increase in new-onset admissions (36 to 69 patients per year). Swift *et al*. [32] followed up 236 T1DM children in Leicestershire aged 10–14 at diagnosis over a 10-year period. The cohort was divided into two groups – home management at diagnosis and hospital management at diagnosis. It was found that the children managed at home initially were less likely to be admitted to hospital in subsequent years than the hospital management group (0.02 per patient-year compared to 0.04 per patient-year). However they were not randomised to home or hospital and so confounding factors cannot be excluded, although the authors found no difference in social background or glycated haemoglobin concentrations between the groups.

A Massachusetts randomised control trial involving 171 patients with insulin-dependent diabetes mellitus (IDDM) "evaluated an ambulatory care intervention aimed at improving glycemic control and reducing hospitalizations [33]." The authors, Laffel and colleagues, reported hospitalisation rates of 0.24 per patient-year for the standard control group of patients and a reduced hospitalisation rate of 0.09 for patients receiving ambulatory diabetes care. In a later publication, Laffel *et al*. [34] carried out another randomised control trial of 123 T1DM children previously diagnosed whilst under the age of 21, comparing sick day management using blood 3-hydroxybutyrate (3-OHB) monitoring with urine ketone monitoring. In this study, DKA hospitalisation rates were reported as 0.75 per patient-year for patients in the urine ketone group, with a lower value of 0.38 per patient-year for the blood ketone group. Although this study was small, it did demonstrate that there is potential for new monitoring programs to facilitate early warning of loss of glycaemic control in T1DM patients and consequent reduction in hospital costs. However, the DKA hospitalisation rates reported were far higher than those reported in other studies where typical rates are between 0.01 and 0.15 per patient-year.

Also in Massachusetts, Levine *et al*. [35] reported overall hospitalisation rates of 0.13 per patient-year in a study assessing three strata of HbA1c levels at baseline for 300 T1DM youths aged 3–16. They commented that this rate was more than three times the rate of 0.04 for all children under 15 years old in the area.

A 2-year prospective, randomised control trial of 299 Boston youths, aged 7–16 years, was conducted by Svoren *et al*. [36] to compare different treatment programmes. One outcome reported was hospitalisation rate for each of the different treatment programmes ranging from 0.09 per patient-year for the group benefiting from psychoeducational modules to 0.13 per patient-year for the standard care group, demonstrating the value of educational programs in reducing costly adverse events.

## Discussion

Most of the publications reviewed related to cohorts in Northern America (16, 50%) or Europe (11, 34%) and the rest related to cohorts in Australasia (5, 16%). For the majority of publications, study design was primarily follow-up of a T1DM cohort over one or more years (18 publications, 56%) or retrospective analysis of hospital admissions over time (8 publications, 25%). In addition, there were two case-control studies with follow-up, three randomised control trials and one review of DKA in T1DM children.

Most of the studies reviewed focussed on DKA admissions (ICD-9 code 250.1) or diabetes-related hospital admissions (ICD-9 codes 250.x) and did not provide any information on other co-morbidities. The only publications to provide data on all hospitalisations were by Icks *et al*. [14,15], Moss *et al*. [6] and Tomlin *et al*. [17] who reported hospitalisation rates as 0.34, 0.27, >0.26 and 0.65 per patient-year, respectively. T1DM hospital admission rates were between three and five times that of the underlying population rates.

Amongst the publications reviewed, DKA hospitalisation rates ranged from 0.01 to 0.18 per patient-year and for diabetes-related hospital admissions, rates ranged from 0.05 to 0.38 per patient-year, excluding the publication by Laffel [34], which reported a very high value of 0.75 per patient-year for the urine ketone monitoring group, primarily because this figure included emergency assessments.

Two publications, Curtis [20] and Donnan, Leese and Morris [16] provided hospital admission rates per 100,000 general population for type 1 cases, but did not provide specific hospitalisation rates for the T1DM cohort, although the latter authors did highlight a three-fold relative risk of re-hospitalisation for type 1 cases compared to the underlying population.

There does not appear to be any fall in admission rates over time following on from the results of the DCCT conducted between 1983 and 1993. There is wide variation in hospitalisation incidence rates reported and some of this variation may be due to the fact that most studies aggregated follow-up observations over several years to obtain an average yearly hospitalisation rate, whereas others only analysed one year of data. One study that did categorise observations into separate years, following initial diagnosis, reported a diabetes-related hospitalisation rate range between 0.07 and 0.10 for different years subsequent to diagnosis [8], but the initial cohort of 577 children had dwindled to 4 children after 8 years, due to "loss to follow-up."

It is evident from the literature that rates of hospitalisation in diabetic children are higher than those observed in the non-diabetic population, with at least a three-fold increase [14-17,35]. In addition, some studies found that duration of each hospital stay for the T1DM cohort was longer than for the general population, by at least a factor of two, further increasing the cost differential [14]. Historically, for procedures which would normally be carried out as day cases, T1DM patients were admitted to hospital several days before surgery for stabilisation, but Robertshaw and Hall [37] report that "More operations are now done on a day-case basis and there is no evidence to support blanket exclusion of diabetic patients from day surgery". However, although time in hospital is reduced, the resource implications for running a diabetic day surgery program are substantial [38].

Several studies found evidence for a link between elevated glycosylated haemoglobin levels (HbA1c) and re-hospitalisation rates. Similarly, there was variation in hospitalisation rates due to age, gender, location and deprivation category. Enhanced psychosocial, psycho-educational and treatment protocol factors reduced overall risk factors for re-hospitalisation. Any reduction in hospitalisation for T1DM children will result in significant healthcare savings, so it is important to identify children most at risk to enable treatment programmes to be targeted for maximum impact.

One of the strengths of the studies in this review is that most started with cohorts of people known to have diabetes. This overcomes the problem of under-reporting of diabetes on hospital discharge records. Williams and colleagues [39] from three English areas reported that the diabetes was not mentioned in 10% of discharge forms when diabetes was the principal cause of admission, and in 23% of forms when the principal reason for admission was closely related to diabetes (for example, amputation). Leslie and colleagues [40] looked at all admissions to all wards of a Scottish teaching hospital, and found that diabetes was omitted in 61% of discharge summaries of patients with the disease.

Diabetes and its complications place a heavy and increasing burden on acute hospitals. Two audits in Liverpool reported that in 1991, 7% of in-patients had diabetes, but by 2003 the figure had risen to 11% [41].

## Conclusion

It is difficult to draw conclusions from the disparate data sets presented here. There is a shortage of studies on hospitalisation patterns in T1DM cases, examining secular trends, yearly admissions, post-diagnosis and analysis of co-morbidity. Information in this area would provide a sound baseline for determination of the efficacy of treatment programmes over time and identification of high risk groups and co-morbidities. Hospitalisation of T1DM children is expensive in terms of healthcare costs and quality of life.

## Competing interests

The author(s) declare that they have no competing interests.

## Authors' contributions

NW and VCA jointly conceived the idea for the review. VCA performed the literature searches and extracted the data. VCA was responsible for analysis and interpretation of the data and approval of the version to be published. VCA drafted the manuscript and NW contributed to revising the article. All authors approved the final version.

## Pre-publication history

The pre-publication history for this paper can be accessed here:


